# Editorial: Patient and medical staff safety and healthy work environment in the 21st century

**DOI:** 10.3389/fpubh.2025.1677117

**Published:** 2025-10-07

**Authors:** Adriano Friganovic, Sandra Boskovic, Sabina Krupa Nurcek, Irena Kovacevic, Justyna Kosydar-Bochenek, Biljana Filipovic

**Affiliations:** ^1^Department of Nursing, University of Applied Health Sciences, Zagreb, Croatia; ^2^Department of Nursing, Faculty of Health Studies, University of Rijeka, Rijeka, Croatia; ^3^Department of Surgery, Faculty of Medicine, Collegium Medicum, University of Rzeszów, Rzeszow, Poland

**Keywords:** patient, medical staff, nurses, healthy work place, work environment

As the 21st century unfolds, bringing rapid technological advances and shifting societal expectations, the healthcare sector faces many challenges and opportunities (1). At the forefront of this transformation lies a dual imperative: ensuring patient safety while simultaneously safeguarding the wellbeing of medical staff (2). A severe shortage of medical staff increases the risk to patient safety and creates an enormous obligation for policymakers and hospital management to work extensively on the creation of a healthy work environment (3). Emotional exhaustion among nurses is a critical factor that significantly impacts patient safety and the overall quality of care in healthcare settings (4).

A healthy, supported, and protected healthcare workforce is the foundation upon which safe, effective, and compassionate patient care is provided (5).

## Understanding safety in a complex healthcare ecosystem

Patient safety has evolved significantly since the publication of the seminal report *To Err Is Human* (6), which brought global attention to the alarming rates of preventable medical errors. Since then, various safety protocols, guidelines, and regulatory frameworks have been introduced (6). However, the complexity of modern healthcare systems means that risks to safety are constantly changing. High patient acuity, staffing shortages, increasing administrative burdens, and rising healthcare demands contribute to a work environment in which both patients and staff are vulnerable (7).

Staff safety has increasingly become a critical concern. According to the World Health Organization, healthcare workers experience some of the highest rates of occupational injury, burnout, and workplace violence (1). The COVID-19 pandemic further underscored these vulnerabilities, revealing significant gaps in emergency preparedness, access to mental health support, and systemic resilience. Whether the goal is ensuring patient wellbeing during medical procedures or safeguarding healthcare workers from occupational hazards, a secure environment is essential for quality care. Table 1 presents key elements of patient and medical staff safety.

**Table 1:** Key elements of patient and medical staff safety.

**Patient safety measures**	**Medical staff safety considerations**
Patients place their trust in medical professionals, expecting care that is free from preventable harm. Some key aspects include:	Healthcare workers operate in high-pressure environments and encounter various risks. Their safety is just as crucial:
- Infection control: hospitals enforce strict hygiene protocols to prevent the spread of diseases.	- Personal Protective Equipment (PPE): shields staff from infections, especially during contagious disease outbreaks.
- Accurate diagnoses and treatments: medical errors can be life-threatening, so making precise assessments and treatments is critical.	- Workplace violence prevention: training and security measures help mitigate the risk of aggression.
- Medication safety: ensuring proper prescriptions, dosages, and patient education minimizes risks.	- Ergonomic workplace design: reducing physical strain prevents long-term health issues.
- Technology and monitoring: advanced systems track patient health in real time, flagging potential complications early.	- Mental health support: stress and burnout are prevalent; support programs ensure staff wellbeing.

## The psychological dimension: burnout and moral injury

Burnout is no longer an isolated issue—it is a widespread occupational syndrome that affects every level of the healthcare workforce (8). Characterized by emotional exhaustion, depersonalization, and a diminished sense of personal accomplishment, burnout compromises clinical decision-making, reduces empathy, and increases the risk of medical errors (9). Compounded by the increasing administrative load and misalignment between organizational goals and personal values, many healthcare professionals have also reported symptoms of *moral injury*—the psychological distress that arises when they are unable to provide the quality of care they know is needed due to systemic constraints (10).

This erosion of the workforce's emotional and psychological health has had a direct impact on patient outcomes. Studies have consistently shown that clinician wellbeing is a strong predictor of patient safety, quality of care, and patient satisfaction.

## Building a culture of safety

A global perspective illustrates how different regions address patient and staff safety. In Europe, the EU-OSHA *Healthy Workplaces Campaign 2023–2025* has emphasized the importance of managing psychosocial risks and promoting digital safety in continental healthcare environments (11). In North America, the Occupational Safety and Health Administration (OSHA) has developed specific guidelines on workplace violence in healthcare, highlighting its impact on staff protection and patient outcomes (12). In Asia, research from Singapore documented high levels of burnout among healthcare workers during the COVID-19 pandemic and identified associated risk factors (13). Complementing this, the WHO Western Pacific Regional Office report underlined the need for sustained investment in workforce protection and capacity building in the post-pandemic era (14). Together, these examples strengthen the global scope of the discussion and illustrate the universal relevance of integrating patient safety with staff wellbeing.

Creating a safe and healthy healthcare environment requires a fundamental shift from individual accountability to systems-based thinking. Healthcare workers must be supported by organizational structures that prioritize safety, transparency, and continuous improvement (15). This includes:

- Leadership commitment to safety as a core organizational value, not just a compliance metric.
- Empowered reporting systems that encourage staff to report near misses and errors without fear of retribution.
- Workplace design improvements, such as ergonomic facilities, adequate rest areas, and reduced noise pollution, all of which enhance focus and reduce stress.
- Adequate staffing levels and skill mix to avoid overwork and ensure proper patient-to-provider ratios.
- Flexible scheduling and leave policies that promote work-life balance and prevent fatigue.

A safety culture must be more than an abstract goal—it must be implemented in daily practice, communication, and organizational policy. It also requires the involvement of all stakeholders, including patients, whose voices can help identify blind spots and improve system responsiveness (15).

## Technology in healthcare

Technology is playing an increasingly prominent role in shaping the healthcare work environment. From electronic health records (EHRs) to AI-enabled decision support systems, digital tools have the potential to reduce errors, improve diagnoses, and streamline care delivery (16).

Healthcare institutions must adopt a human-centered approach to technology implementation. This involves engaging clinicians in the design and testing of digital tools, ensuring proper training, and continuously monitoring the impact of technology on both staff workload and patient outcomes.

As healthcare systems increasingly rely on interconnected digital platforms, cybersecurity and data privacy must be integral components of safety strategies. Ensuring the confidentiality, integrity, and availability of patient information protects both patients and staff from emerging digital threats (17). Alongside these benefits, however, research also points to risks associated with digitalization. Work-related technologies can contribute to technostress, blurred boundaries between work and private life, and increased psychosocial strain on staff, as highlighted in a recent scoping review of the public sector (18). At the same time, other evidence confirms that the adoption of digital health technologies can significantly improve staff performance while reducing workload, particularly in resource-constrained hospital environments (19).

## Occupational health: from reactive to preventive models

Occupational health in healthcare settings has often been reactive, focusing on injury management and infection control. A 21st-century approach must be preventive and holistic. Insights from other sectors also demonstrate the value of digital technologies—systematic reviews highlight how wearables, sensors, and AI can transform occupational health by enabling real-time monitoring and prevention strategies (20). This includes:

- Regular mental health screenings and support services.
- Promotion of healthy lifestyle behaviors through institutional wellness programs.
- Measures to prevent workplace violence, including training in de-escalation and physical security.
- Access to vaccinations and personal protective equipment as standard protocol.

In addition, the introduction of digital technologies into daily workflows has been associated with psychosocial challenges such as technostress and work–life imbalance, as highlighted by Håkansta et al. (18). These findings emphasize that occupational health strategies must address not only physical but also digital and psychosocial risks. Emerging Industry 4.0 innovations—such as drones, collaborative robots, wearable sensors, and VR/AR-based training—are increasingly being applied to strengthen occupational safety and health systems (21). These technologies provide new opportunities for proactive monitoring, prevention, and staff training.

Health systems must also acknowledge the long-term effects of traumatic experiences, especially among emergency and critical care workers (22). Providing access to trauma-informed care and peer support programs is essential to maintaining a resilient workforce. Incorporating digital health technologies has also been shown to ease workload pressures and enhance efficiency among healthcare staff, thereby contributing to preventive occupational health strategies (19).

## Equity and inclusion as safety imperatives

The health and safety of healthcare environments are deeply linked to equity and inclusion. Marginalized staff often experience higher levels of discrimination, abuse, microaggressions, and psychological distress (23–26).

By embedding equity into safety strategies—through bias training, inclusive hiring practices, and patient-centered communication—healthcare organizations can address both overt and subtle risks to wellbeing.

## Conclusion: the path forward

Patient safety and staff wellbeing must be addressed as one system-level priority. Safe healthcare environments require collaboration across professions and sustained investment in education, innovation, and human resources. The COVID-19 pandemic demonstrated globally—from Europe to North America and Asia—that unprepared systems face higher rates of burnout, errors, and loss of trust. The cost of neglect extends beyond preventable harm to diminished public confidence. Aligning staff wellbeing with patient safety is essential to ensuring effective, compassionate, and resilient healthcare.
